# Eco-Friendly Fabrication of Highly Stable Silica Aerogel Microspheres with Core–Shell Structure

**DOI:** 10.3390/polym15081882

**Published:** 2023-04-14

**Authors:** Gao Cai, Haisong Ni, Xunzhang Li, Yangxin Wang, Huaixia Zhao

**Affiliations:** College of Materials Science and Engineering, Nanjing Tech University, 30 South Puzhu Road, Pukou District, Nanjing 211816, China

**Keywords:** aerogel spheres, core–shell structures, silicas, polysiloxanes, thermal insulation

## Abstract

Silica aerogel microspheres show great potential in various fields as fillings in different materials. It is important to diversify and optimize the fabrication methodology for silica aerogel microspheres (SAMS). This paper presents an eco-friendly synthetic technique for producing functional silica aerogel microspheres with a core–shell structure. Mixing silica sol with commercial silicone oil containing olefin polydimethylsiloxane (PDMS) resulted in a homogeneous emulsion with silica sol droplets dispersed in the oil. After gelation, the droplets were transformed into silica hydrogel or alcogel microspheres and coated with the polymerization of the olefin groups. Microspheres with silica aerogel as their core and polydimethylsiloxane as their shell were obtained after separation and drying. The sphere size distribution was regulated by controlling the emulsion process. The surface hydrophobicity was enhanced by grafting methyl groups onto the shell. The obtained silica aerogel microspheres have low thermal conductivity, high hydrophobicity, and excellent stability. The synthetic technique reported here is expected to be beneficial for the development of highly robust silica aerogel material.

## 1. Introduction

Silica aerogels are one kind of mesoporous amorphous material with many distinctive characteristics, such as low bulk density, low thermal conductivity, low refractive index, high porosity, and high specific surface area [1,2,3,4,5], which are derived from the nanoporous network of interconnected primary particles. The voids in the network structure of silica aerogels account for more than 90% of the whole volume. Although silica aerogels are widely used in aerospace vehicles [6,7,8], civil buildings [9,10], clothes [11,12], environmental purification materials [13], and drug delivery systems [14,15,16,17], their characteristics that are not conducive to ductility lead to poor mechanical properties for silica aerogel monoliths. This makes their direct use or hybridization with other materials difficult, which seriously limits the application of silica aerogel in many fields [18,19,20,21]. Moreover, the repeated modification process to transfer hydrophilicity to the hydrophobic surface of the silica aerogel monolith is tedious and requires an extremely long processing time [22].

Instead, silica aerogel powders, e.g., microspheres or granules, as a new product form, can make up for the poor mechanical performance of silica aerogel bulk when used as fillings hybridized with other materials [23,24,25,26,27]. In addition, due to the tiny volume of the silica aerogel powders, the modification and drying process is easier and faster, which effectively decreases the fabrication time. A few silica aerogel powders have been recently reported with various methods for rapid synthesis [22]. Pan et al. reported a method to synthesize superhydrophobic (θ = 162°) aerogel powders at ambient pressure using a water-glass-based sol–gel method with a technological process of mechanical crushing and filtration [28]. Bhagat et al. reported superhydrophobic silica aerogel powders with simultaneous surface modification, solvent exchange, and sodium ion removal from hydrogels [29]. Lee et al. reported a fast synthesis technique for silica aerogel powders by an emulsion polymerization method using water glass, n-hexane, and a surfactant (Span80) [30]. Jiang et al. prepared an amine-grafted silica aerogel microsphere by dropping the siliceous solution into a hot oil bath [31].

Although these methods successfully produced different SiO_2_ aerogel powders, there are still some challenges, such as the imprecise control of the aerogel powder size in the method of mechanical crushing and the one-step synergetic fabrication, and the strong dependence on volatile and poisonous organic solvents in the emulsion method. The method of dropping the siliceous solution into a hot oil bath is environmentally friendly but only suitable for the preparation of big gel microspheres with a size in the millimeter scale. It is thus still urgent to develop new methods for the efficient fabrication of silica aerogel powders. The stability of the silica aerogel powders is another important challenge for the development of the silica aerogel. Undecorated silica aerogel can be easily destroyed once immersed in organic or inorganic solvents due to the high void volume fraction and the relatively weak physical interaction between the nanoparticles. Most of the currently reported silica aerogel spheres are modified with hydrophobic groups, which can efficiently prevent water invasion to nanoporous structures. However, when these hydrophobic silica aerogel spheres make contact with organic solvents, the nanoporous structures are filled and destroyed. To date, very few studies involve silica aerogel spheres that are stable in both water and organic solvents.

In this study, an eco-friendly and facile synthetic technique is reported that produces functional silica aerogel microspheres (SAMS) with a core–shell structure. Droplets of silica sol containing a gelation catalyst and polymerization initiators were obtained by mixing silica sol with commercial silicone oil and olefin polydimethylsiloxane (PDMS) under stirring. After gelation, the droplets were transformed into silica hydrogel or alcogel in the form of microspheres, which were further coated by the subsequent polymerization of the olefin groups on the polydimethylsiloxane. After separation and drying, the core–shell silica aerogel microspheres were obtained. This method has the following advantages: 1. The size distribution of the core–shell silica aerogel microspheres can be effectively regulated by controlling the ratios of the silica sol to the silicone oil in the emulsion procedure. 2. The obtained microspheres with silica aerogel as their core and PDMS as their shell show excellent stability in both water and organic solvents. 3. Various silica sources can be used in the reported synthetic technique, e.g., water glass and TEOS. 4. The reported synthetic technique uses silicone oil as the fabrication medium, which is environmentally friendly. The specific synthetic technique reported here is expected to be beneficial for the development and application of highly robust silica aerogels.

## 2. Materials and Methods

### 2.1. Materials and Methods

Water glass (WG) was supplied by Quechen Silicon Chemical Co., Ltd., Wuxi, China. Glacial acetic acid (HAc), tetraethyl orthosilicate (TEOS), ammonia solution (AR), sodium dodecyl sulfate (SDS), and ethanol absolute (EtOH) was purchased from Sinopharm Chemical Reagent Co., Ltd., Nanjing, China. Silicone oil PMX-200 (10 mPa.s) and triethoxymethylsilane (MTES) were purchased from Shanghai Aladdin Biochemical Technology Co., Ltd., Shanghai, China. Hydrochloric acid (HCl) was purchased from Yonghua Chemical Co., Ltd., Suzhou, China. Olefin dimethylsiloxane (5000 mPa.s) was purchased from Dow Chemical Company, Midland, MI, USA. Potassium persulfate (KPS) was purchased from Beijing InnoChem Science & Technology Co., Ltd., Beijing, China. 2,2′-Azobis (2-methylpropionitrile) (AIBN) was purchased from Shanghai Macklin Biochemical Co., Ltd., Shanghai, China. DI water (W) was home-made. All chemicals were used as received without any further purification. The morphology and particle size of the silica aerogel microspheres (SAMS) were investigated using a scanning electron microscope (SEM, ZEISS 1530VP, Carl Zeiss Microscopy Ltd., Cambridge, UK). Specific surface area and pore size distribution were characterized by N_2_ adsorption/desorption test (BET, Quantachrome Autosorb-iQ analyzer, Quantachrome Instruments, Boynton Beach, FL, USA). The core–shell structure of the SAMS was studied using a fluorescence microscope (Nreeohy Y-E68, Shenzhen Nreeohy Technology Co., Ltd., Shenzhen, China). The contact angle was measured using a contact angle meter (JC2000C, Shanghai Zhongchen Digital Technology Equipment Co., Ltd., Shanghai, China). In order to better test the contact angle of aerogel particles, the aerogel particles were compressed to the block plates with certain thicknesses and relatively flat surfaces. The specimen was put on the instrument, and 5 μL deionized water was dropped on the surface with a syringe at room temperature, and then we determined the contact angle with a graphic method.

### 2.2. Fabrication of wg-SAMS-4.5/3.5/2.5

WG, W, and HAc were mixed with a mass ratio of 1.35:5:0.357 under room temperature (25 °C) to form a water glass solution, which was then dropped into the mixture of silicone oil and olefin polydimethylsiloxane (PDMS) under strong mechanical stirring to form the homogenous emulsion. The mass ratio of the water glass solution, olefin polydimethylsiloxane, and silicone oil was 4.5:1:1, 3.5:1:1, and 2.5:1:1 for the samples of wg-SAM-4.5/3.5/2.5, respectively. The obtained emulsions were then gelated at room temperature (25 °C) for 24 h to obtain spherical wet silica gels dispersed in the silicone oil. Silica gel spheres were then washed with silicone oil, 2% SDS aqueous solution, and ethanol (EtOH) with centrifugation, and then aged in EtOH for 48 h. The silica gel microspheres were then dried with freeze–vacuum drying to obtain the SiO_2_ aerogel of wg-SAMS-4.5/3.5/2.5.

### 2.3. Fabrication of teos-SAMS-4.5/3.5/2.5

TEOS, EtOH, W, and HCl were mixed with a mass ratio of 2.632:6.314:0.9:0.044 at 50 °C for 1 h to form a siliceous solution, into which was added a mass ratio of 0.136 of ammonia solution (2 wt% in ethanol). The obtained siliceous solution was then dropped into the mixture of silicone oil and olefin polydimethylsiloxane (PDMS) under strong mechanical stirring to form the homogenous emulsion. The mass ratio of the siliceous solution, olefin silicone oil, and silicone oil was 4.5:1:1, 3.5:1:1, and 2.5:1:1. The obtained emulsions were then gelated at room temperature (25 °C) for 24 h to obtain spherical wet silica gels dispersed in the silicone oil. Silica gel spheres were then washed with silicone oil, 2 wt% SDS aqueous solution, and EtOH with centrifugation, and then aged in EtOH for 48 h. The silica gel microspheres were then dried with freeze–vacuum drying to obtain the SiO_2_ aerogel of teos-SAMS-4.5/3.5/2.5.

### 2.4. Fabrication of wg-SAMS-4.5@PDMS

WG, W, KPS, and HAc were mixed with a mass ratio of 1.35:5:0.36:0.357 under room temperature (25 °C) to form a water glass solution, which was then dropped into the mixture of olefin silicone oil and silicone oil under strong mechanical stirring to form the homogenous emulsion. The mass ratio of the water glass solution, olefin silicone oil, and silicone oil was 4.5:1:1. The obtained emulsions were then gelated at room temperature (25 °C) for 24 h to obtain spherical wet silica gels dispersed in the olefin silicone oil. The emulsion containing silica gel microspheres was then heated at 60 °C for 8 h for polymerization of the olefin silicone oil forming polydimethylsiloxane (PDMS) coating on the surface of the silica gel spheres, which were then washed with silicone oil, 2 wt% SDS aqueous solution, and EtOH with centrifugation, and then put in EtOH for 48 h. The PDMS-coated silica gel microspheres were dried by freeze–vacuum drying to form the PDMS-wrapped SiO_2_ aerogel of wg-SAMS-4.5@PDMS.

### 2.5. Fabrication of teos-SAMS-4.5@PDMS

TEOS, EtOH, W, AIBN, and HCL (2 wt% in ethanol) were mixed in a mass ratio of 2.632:6.314:0.9:0.48:0.044 at 50 °C for 1 h to form a siliceous solution, to which was added a mass ratio of 0.136 ammonia solution (2% in ethanol) at room temperature. The obtained siliceous solution was then dropped into the mixture of silicone oil and olefin silicone oil under strong mechanical stirring to form the homogenous emulsion. The mass ratio of the siliceous solution, olefin silicone oil, and silicone oil was 4.5:1:1. The obtained emulsions were then gelated at room temperature (25 °C) for 24 h to obtain spherical wet silica gels dispersed in the silicone oil. The emulsion containing silica gel microspheres was then heated at 80 °C for 10 h for polymerization of olefin silicone oil forming PDMS coating on the surface of the silica gel spheres, which were then washed with silicone oil, 2 wt% SDS aqueous solution, and EtOH with centrifugation, and then put in EtOH for 48 h. The PDMS-coated silica gel microspheres were dried by freeze–vacuum drying to form the PDMS-wrapped SiO_2_ aerogel of teos-SAMS-4.5@PDMS.

### 2.6. Fabrication of wg-SAMS-4.5@PDMS-Me

This fabrication process was similar to the fabrication of wg-SAMS-4.5@PDMS; the only difference was that after the PDMS coating, MTES with a mass ratio of 7.16 was added to the mixture, which was heated at 60 °C for an extra 6 h. The silica gel spheres were then washed with silicone oil, 2% SDS aqueous solution, and EtOH with centrifugation, and then put in EtOH for 48 h. The modified silica gel microspheres were dried by freeze–vacuum drying to form the wg-SAMS-4.5@PDMS-Me.

### 2.7. Fabrication of teos-SAMS-4.5@PDMS-Me

This fabrication process was similar to the fabrication of teos-SAMS-4.5@PDMS; the only difference was that after the PDMS coating, MTES, W, and HCl (2 wt% in ethanol) with a mass ratio of 8.95:10:5.5 were added to the mixture which was then heated at 60 °C for another 6 h. The silica gel spheres were then washed with silicone oil, 2 wt% SDS aqueous solution, and EtOH with centrifugation, and then put in EtOH for 48 h. The modified silica gel microspheres were dried by freeze–vacuum drying to form the teos-SAMS-4.5@PDMS-Me.

## 3. Results and Discussion

**Figure 1:**
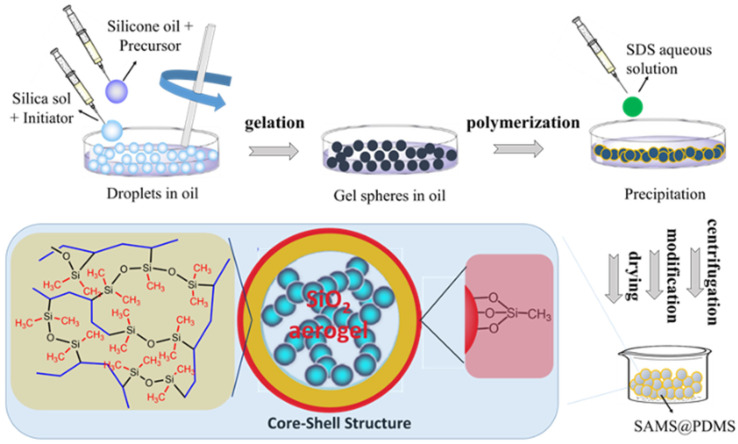
Schematic representation of the fabrication process and the core–shell structure of the PDMS-coated SiO_2_ aerogel microspheres (SAMS@PDMS).

The fabrication of the silica aerogel microspheres coated with polydimethylsiloxane (SAMS@PDMS) is presented in Figure 1. The whole process mainly includes micellization, gelation, polymerization, and modification. Two kinds of cheap silicon sources, water glass and tetraethyl orthosilicate (TEOS), were used to obtain the initial silica sol. When dropping these two silica sols into the mixture of alkylene polydimethylsiloxane (PDMS) and silicone oil under fierce stirring, silica sol droplets in the shape of microspheres formed in the obtained water–oil emulsion. The regulation of the size distribution of the formed silica sol droplets was explored by changing the ratios of the silica sol in the silicone oil (Figure S1). Figure S2 summarizes the size distribution of the silica sol droplets with the varying amount of silica sol in the silicone oil during the fabrication process. The results show that the size of the silica sol microspheres is significantly affected by the ratios of the silica sol in the dispersion phase. As the ratios of the silica sol in the silicone oil decrease, the viscosity of the mixture emulsion decreases, which makes possible the existence of silica sol microspheres with a large size. In addition, the choice of the silicon sources could also obviously influence the size distribution of the silica sol microspheres in the emulsion stage. The size of the microspheres of the silica sol with TEOS as the silica source is obviously larger than with water glass as the silica source. Moreover, increasing the stirring time could homogenize the size distribution of the silica sol microspheres in the emulsion.

**Figure 2:**
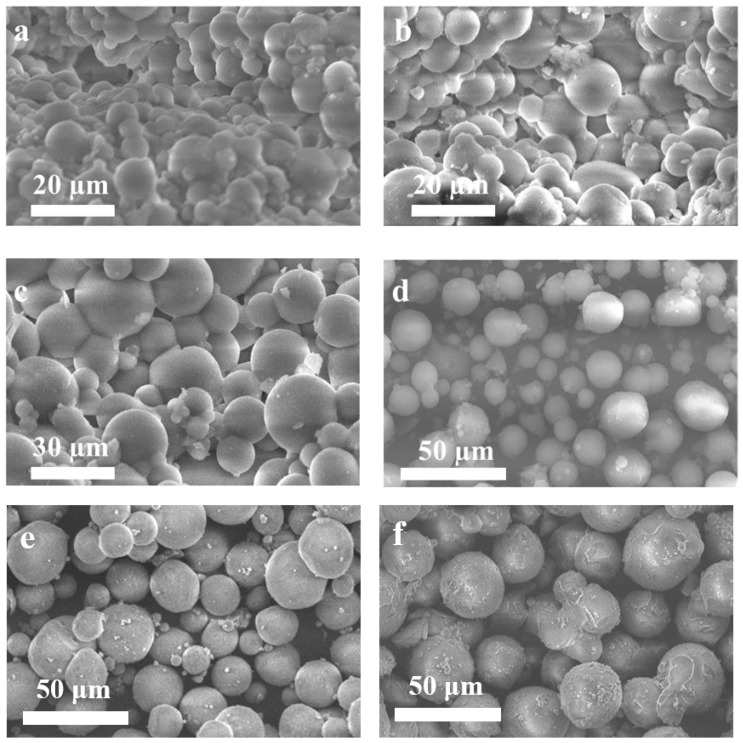
SEM images of pure silica aerogel spheres of wg-SAMS-4.5 (**a**), wg-SAMS-3.5 (**b**), wg-SAMS-2.5 (**c**), teos-SAMS-4.5 (**d**), teos-SAMS-3.5 (**e**), and teos-SAMS-2.5 (**f**).

We further explored the fabrication process and investigated the critical influencing factors in detail step by step. After the full gelation process, the obtained wet gel microspheres were observed in situ under an optical microscope. As shown in Figures S3 and S4, the wet gel microspheres have almost the same size distribution as that of the above-mentioned silica sol. This demonstrates that during the gelation process the size and the shape of the microspheres in the emulsion are well retained. The emulsion mixture containing the wet gel spheres was then demulsified with SDS solution, followed by washing, centrifugation, and freeze–vacuum drying, resulting in the pure aerogel SAMS. The SEM images of SAMS prepared from different ratios of silica sol in silicone oil are shown in Figure 2. The regulation of the size distribution of the silica aerogel microspheres shows the same variation trend as that of the wet gel microspheres with varying ratios of the silica sol in silicone oil during the fabrication process. When a 4.5 mL solution of water glass was added to the dispersion phase of 1 mL of silicone oil in the fabrication process, the size of the resulting SiO_2_ aerogel sphere was about 5 µm (Figure 2a). Keeping the volume of the dispersion phase of the silicone oil unchanged, when the volume of the water glass solution decreased to 3.5 mL and 2.5 mL, the size of the resulting SiO_2_ aerogel sphere increased to about 8 µm and 15 µm, respectively (Figure 2b,c). It was clearly observed that the mass ratio of the continuous phase and the dispersion phase is the most important influence factor for the size distribution of the resulting aerogel sphere. In addition, with the same ratios of the silica sol in silicone oil in the fabrication process, the size of the aerogel microspheres with TEOS as the silica source was obviously larger than with water glass as the silica source. When a 4.5 mL solution of TEOS was added to the dispersion phase of 1 mL of silicone oil in the fabrication process, the size of the resulting SiO_2_ aerogel sphere was about 14 µm (Figure 2d). Keeping the volume of the dispersion phase of the silicone oil unchanged, when the volume of the TEOS solution decreased to 3.5 mL and 2.5 mL, the size of the resulting SiO_2_ aerogel sphere increased to about 19 µm and 26 µm, respectively (Figure 2e,f). The same size regulation rule between the silica sol droplets and the SiO_2_ aerogel spheres shows that the size of the aerogel microspheres could be regulated by controlling the formation process of the emulsion. Moreover, the size distribution of the wet gel microspheres and the corresponding aerogel microspheres is similar, which means that the microstructure of the SiO_2_ nanoparticles is well retained during the drying process (Figures S4 and S5).

N_2_ adsorption–desorption isotherms of the SiO_2_ aerogel microspheres are shown in Figure S6. The isotherms for SAMS display type IV isotherms with H1 hysteresis loops. The noticeable saturation adsorption platforms demonstrate that there are no macropores in the SAMS, which is supported by their pore size distribution curves. The pore size distribution curves of the SAMS also show that the pores are concentrated in the 2–15 nm range. The results demonstrate that the silica source used in the fabrication process has the most obvious influence on the nanostructures of the aerogel microspheres. The specific surface areas of all three samples of wg-SAMS-4.5/3.5/2.5 obtained with water glass as the silica source in fabrication are more than 970 m^2^g^−1^, which are obviously larger than the surface areas of the samples of teos-SAMS of 745.49 m^2^g^−1^, which were obtained with TEOS as the silica source. Similarly, the mean pore diameters of SAMS with different silica sources are also different (Table S1).

In order to obtain the core–shell structure, the initiators were induced into the emulsion system during the fabrication process to trigger the polymerization of the alkylene group in the polydimethylsiloxane framework, which was coated on the surface of the microspheres of the wet silica gel. For the different silicon sources, water glass and TEOS, the inorganic and organic initiators potassium persulfate and 2,2′-azobis (2-methylpropionitrile) (AIBN) were used, respectively. AIBN and potassium persulfate were added to the alcohol solution of TEOS and the aqueous solution of water glass, respectively. After the gelation process, the emulsion mixtures were heated to trigger the polymerization of olefin polydimethylsiloxane, thus coating the wet silica gel spheres and forming the core–shell structure. After the subsequent demulsification, washing, and separation by centrifugation and freeze–vacuum drying, the SiO_2_ aerogel microspheres of wg-SAMS-4.5@PDMS and teos-SAMS-4.5@PDMS were obtained.

**Figure 3:**
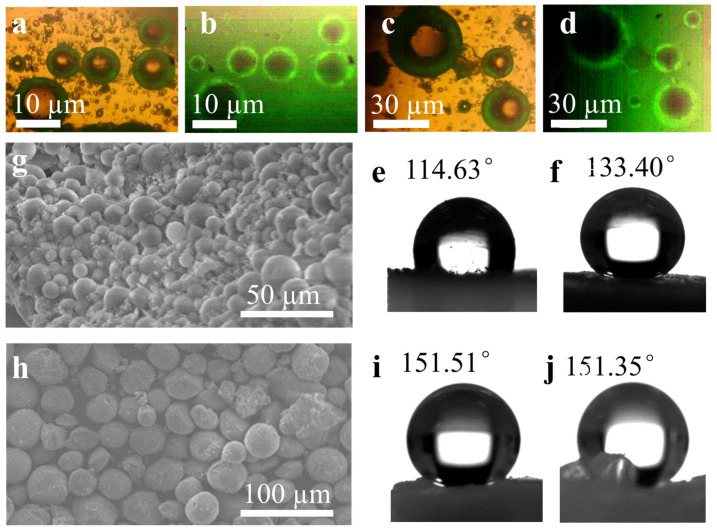
Optical microscope images of the core–shell structure of PDMS-wrapped SiO_2_ aerogel microspheres of wg-SAMS-4.5@PDMS (**a**,**b**) and teos-SAMS-4.5@PDMS (**c**,**d**) under white light (**a**,**c**) and blue light (**b**,**d**) (480 nm), respectively. Photographs of water droplets on the samples of wg-SAMS-4.5@PDMS (**e**) and teos-SAMS-4.5@PDMS (**f**). SEM images of methyl-modified PDMS-wrapped SiO_2_ aerogel microspheres of wg-SAMS-4.5@PDMS-Me (**g**) and teos-SAMS-4.5@PDMS-Me (**h**). Photographs of water droplets on the samples of wg-SAMS-4.5@PDMS-Me (**i**) and teos-SAMS-4.5@PDMS-Me (**j**).

Fluorescence optical microscopy was used to explore the core–shell structure of the PDMS-wrapped silica aerogel microspheres of wg-SAMS-4.5@PDMS and teos-SAMS-4.5@PDMS. Before the fluorescent imaging, these two PDMS-wrapped silica aerogel microspheres were immersed in an aqueous solution of a fluorescent dye for a few days to let the dye molecules diffuse into the PDMS coating completely. The chemical structure of the fluorescent dye was a water-soluble sodium salt of N,N′-Di (2-succinic acid)-perylene-3,4,9,10-tetracarboxylic bisimide. The perylene structure made it compatible with the polydimethylsiloxane (PDMS) network but incompatible with the inorganic silica. Therefore, the dye molecule could pass through the PDMS matrix and stain the shell structure, separating the PDMS shell from the silica core in the imaging process. As shown in Figure 3, these two samples of wg-SAMS-4.5@PDMS and teos-SAMS-4.5@PDMS show as solid spheres under white-light irradiation (Figure 3a,c), while obvious core–shell structures were observed under irradiation with a blue light of 480 nm (Figure 3b,d). It is difficult for the dye molecule to enter inorganic SiO_2_ aerogels, so there is no fluorescence emission even under the irradiation of blue light. The PDMS shell can be clearly distinguished from the SiO_2_ aerogel core because of the fluorescence emission. The formation of the PDMS shells on the SiO_2_ aerogel core was further demonstrated by the FT-IR spectra analysis (Figure S7).

The special framework of PDMS also influences the hydrophobicity of the SAMS@PDMS samples. As shown in Figure 3e,f, the water contact angles of wg-SAMS-4.5@PDMS and teos-SAMS-4.5@PDMS are 114.63° and 133.40°, respectively. The hydrophobicity is just on the middle level compared with the superhydrophobic material. This is due to the obvious hydrophilicity of the Si-O-Si in the main chain of PDMS, which decreases the hydrophobicity of the methyl group on the side chain of PDMS. In order to enhance the hydrophobicity performance of the SAMS@PDMS, further methylation was performed on the PDMS coating. During the fabrication procedure, after the polymerization of the olefin polydimethylsiloxane on the surface of the SiO_2_ wet gel, the coated SiO_2_ sphere was separated by centrifugation. The obtained SiO_2_ aerogel sphere was then dispersed in the ethanol solution of MTES to modify the PDMS surface with the methyl group. The obtained wg-SAMS-4.5@PDMS-Me and teos-SAMS-4.5@PDMS-Me both maintained their sphere shape very well (Figure 3g,h). Notably, the hydrophobicity of these two methyl-modified samples was obviously enhanced to superhydrophobicity levels with a more than 150° water contact angle (Figure 3i,j).

**Figure 4:**
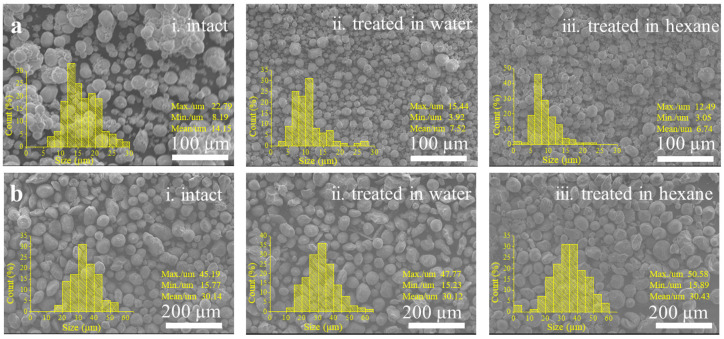
(**a**) SEM images and the size distribution statistics data of the pure SiO_2_ aerogel microspheres of teos-SAMS-4.5 at different states: intact sample (**i**), dried sample after immersion in water for 5 days (**ii**), and dried sample after immersion in hexane for 5 days (**iii**). (**b**) SEM images of the PDMS-wrapped silica aerogel microspheres of teos-SAMS-4.5@PDMS at different states: intact sample (**i**), dried sample after immersion in water for 5 days (**ii**), and dried sample after immersion in hexane for 5 days (**iii**). Insets show the size distribution of the aerogel microspheres.

The porous nanostructure of the silica aerogel endows it superthermal insulation. However, the pure SiO_2_ aerogel is usually sensitive to solvents. Once the solvents enter the pores, the evaporation of the solvents can destroy the nanostructure which is composed of connected SiO_2_ particles, making the related material lose its thermal insulation. In our study, water and hexane were chosen to estimate the stability of the SAMS and SAMS@PDMS to organic and inorganic solvents, respectively. As shown in Figure 4a, when we put the pure SiO_2_ aerogel sample of teos-SAM-4.5 in water and in hexane for 5 days, the sizes of the aerogel microspheres in both cases sharply reduced, which is caused by the destruction of the porous nanostructures during the immersion and drying process. Notably, when the PDMS-wrapped aerogel sample of teos-SAMS-4.5@PDMS was put in water and in hexane, the SiO_2_ aerogel maintained its sphere shape very well with unchanged size distribution (Figure 4b).

**Figure 5:**
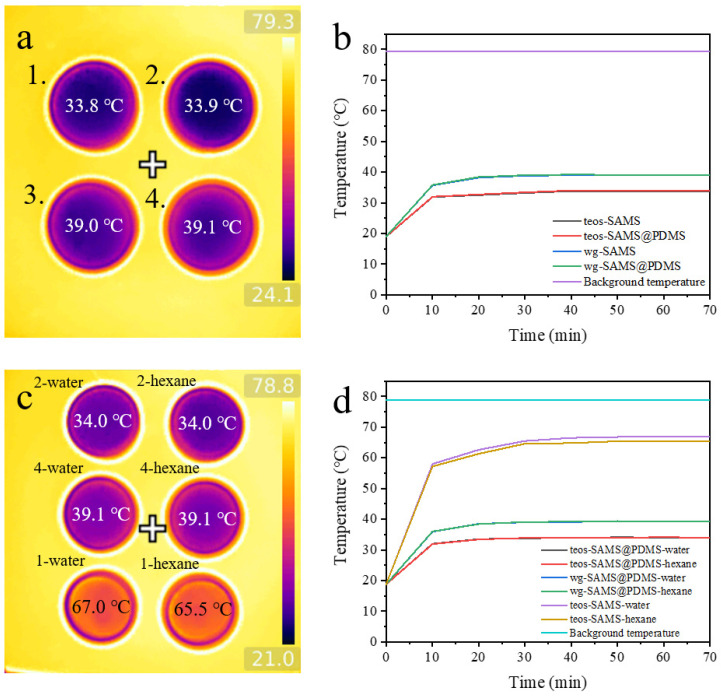
(**a**) Infrared images of the intact powders of teos-SAMS-4.5 (1), teos-SAMS-4.5@PDMS (2), wg-SAMS-4.5 (3), and wg-SAMS-4.5@PDMS (4) on the hot surface. (**b**) The surface temperature curve as a function of time for SAMS and SAMS@PDMS. (**c**) Infrared images of the solvent-treated powders of teos-SAMS-4.5 (1-water and 1-hexne), teos-SAMS-4.5@PDMS (2-water and 2-hexane), and wg-SAMS-4.5@PDMS (4-water and 4-hexane) on the hot surface. 1-water and 1-hexne: teos-SAMS-4.5 powder after immersion in water and in hexane for 5 days, respectively; 2-water and 2-hexne: teos-SAMS-4.5@PDMS powder after immersion in water and in hexane for 5 days, respectively; 4-water and 4-hexne: wg-SAMS-4.5@PDMS powder after immersion in water and in hexane for 5 days, respectively. During the thermal insulation test, the aerogel powders were put in glass petri dishes without lids, which were put on the hot face for 70 min before imaging. (**d**) Surface temperature curve as a function of time for solvent-treated SAMS and SAMS@PDMS.

In order to explore the protective effectiveness of the PDMS shell for the nanoporous structure of the SiO_2_ aerogel powders, we compared the thermal insulation performance of the pure SiO_2_ aerogel samples and the PDMS-wrapped aerogel samples before and after the solvent treatment. Before the solvent treatment, the thermal conductivity of teos-SAMS and teos-SAMS@PDMS was 0.01976 (W/m·k) and 0.01985 (W/m·k), respectively. The thermal conductivity of wg-SAMS and wg-SAMS@PDMS was 0.02105 (W/m·k) and 0.02188 (W/m·k), respectively. As shown in Figure 5a,b, the intact samples of teos-SAMS-4.5 and teos-SAMS-4.5@PDMS have similar thermal insulation, as do the samples of wg-SAMS-4.5 and wg-SAMS-4.5@PDMS, which demonstrates that the PDMS shell does not decrease the thermal insulation of the aerogel particles. We then compared the recycled teos-SAMS-4.5, wg-SAMS-4.5@PDMS, and teos-SAMS-4.5@PDMS at the same heating state for 70 min to test their thermal insulation performance. Figure 5c,d show that the thermal insulation of the recycled aerogel samples of teos-SAM-4.5 decreases obviously compared with the intact sample, which is supposed to raise from the breaking down of the porous structure of the aerogel. After treatment with water and n-hexane, the thermal conductivity of teos-SAMS was 0.08437 (W/m·k) and 0.06285 (W/m·k), respectively, while the thermal conductivity of wg-SAMS@PDMS and teos-SAMS@PDMS remained unchanged. Notably, the recycled PDMS-wrapped aerogel samples of teos-SAMS-4.5@PDMS and wg-SAMS-4.5@PDMS can retain the same thermal insulation level as that of the intact sample, which demonstrates that the PDMS shell could effectively help maintain the integrity of the porous structure of the SiO_2_ aerogel core.

## 4. Conclusions

In summary, we have reported a facile synthetic technique to produce functional silica aerogel microspheres with a core–shell structure. Water glass and tetraethyl orthosilicate were used as the precursors of the silica aerogels. The silica sol was firstly gradually added into the mixture of commercial silicone oil and olefin polydimethylsiloxane under strong stirring, resulting in a homogeneous emulsion with droplets of silica sol in the silicone oil. During the subsequent sol–gel process, the droplets of silica solution were transformed into hydrogel or alcogel microspheres, which were further coated by the subsequent polymerization of the olefin groups on the polydimethylsiloxane. After centrifugal separation and freeze–vacuum drying, the core–shell microspheres were obtained. The size distribution of the core–shell silica aerogel microspheres was effectively regulated. The surface hydrophobicity was further enhanced by grafting the methyl group onto the shell of the silica aerogel microspheres. The obtained silica aerogel microspheres have low thermal conductivity, superhydrophobicity, and excellent stability. The reported synthetic technique is expected to offer a new fabrication strategy for highly robust silica aerogel.

## Data Availability

Data will be made available on request.

## Supplementary Materials

The following supporting information can be downloaded at: https://www.mdpi.com/article/10.3390/polym15081882/s1, Figure S1: optical images of the silica sol microdroplets; Figure S2: size distribution of silica sol microdroplets; Figure S3: optical images of the silica wet gel microspheres; Figure S4: size distribution of silica wet gel microspheres; Figure S5: size distribution of silica aerogel microspheres; Figure S6: adsorption–desorption isotherms of silica aerogel microspheres; Figure S7: FTIR spectra curves of aerogel specimens; Table S1: physical properties of silica aerogel microspheres of wg-SAMS-4.5, wg-SAMS-3.5, wg-SAMS-2.5, and teos-SAMS-4.5.
