# Supplementary material for: Eco-Friendly Fabrication of Highly Stable Silica Aerogel Microspheres with Core–Shell Structure
Source: Polymers (Basel). 2023 Apr 14;15(8):1882. doi: 10.3390/polym15081882 (PMC10146462; doi:10.3390/polym15081882)
Supplement: Supplementary file 1 [file polymers-15-01882-s001.zip › polymers-2270936-supplementary.pdf]

Supporting Information

Eco-Friendly Fabrication of Highly Stable Silica Aerogel Microspheres with Core–Shell Structure

Gao Cai, Haisong Ni, Xunzhang Li, Yangxin Wang * and Huaixia Zhao *

College of Materials Science and Engineering, Nanjing Tech University, 30 South Puzhu Road, Pukou District, Nanjing 211816, China

* Correspondence: yangxin.wang@njtech.edu.cn (Y.W.); zhaohx@njtech.edu.cn (H.Z.).

S1. Optical images and size distribution

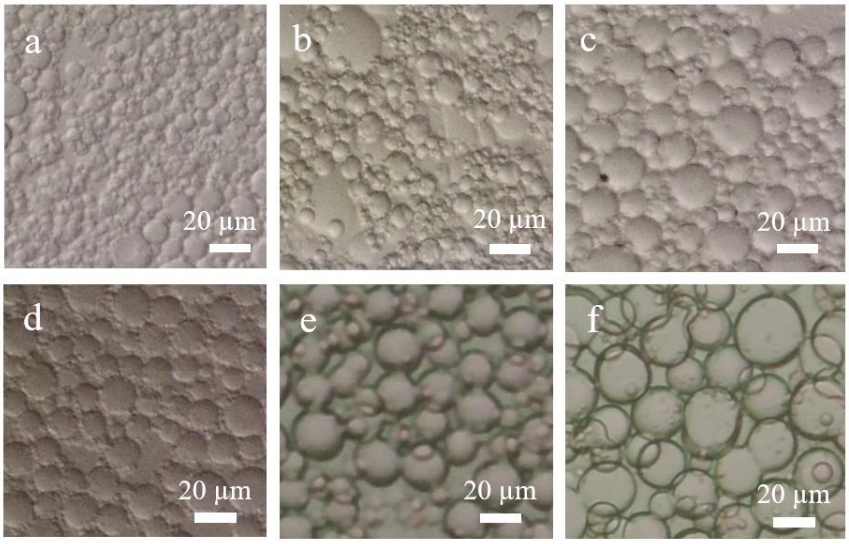

Figure. S1 Optical images of the silica sol micro droplets of wg-SAMS-4.5-droplet (a), wg-SAMS-3.5-droplet (b), wg-SAMS-2.5-droplet (c), teos-SAMS-4.5-droplet (d), teos-SAMS-3.5-droplet (e), and teos-SAMS-2.5-droplet (f).

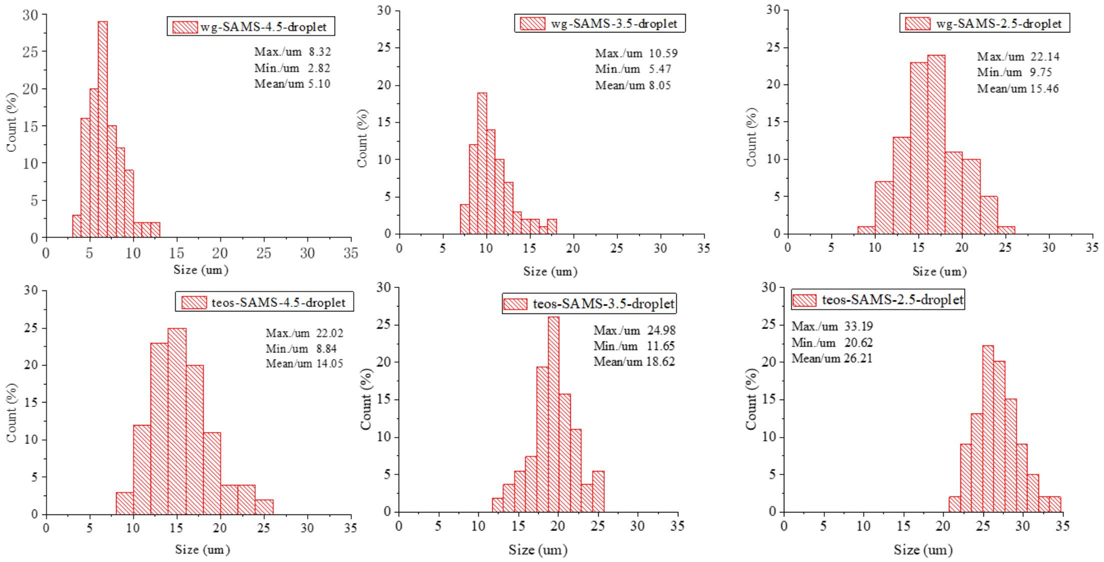

Figure. S2 Size distribution of silica sol micro droplets with different sol contents in silicone oil.

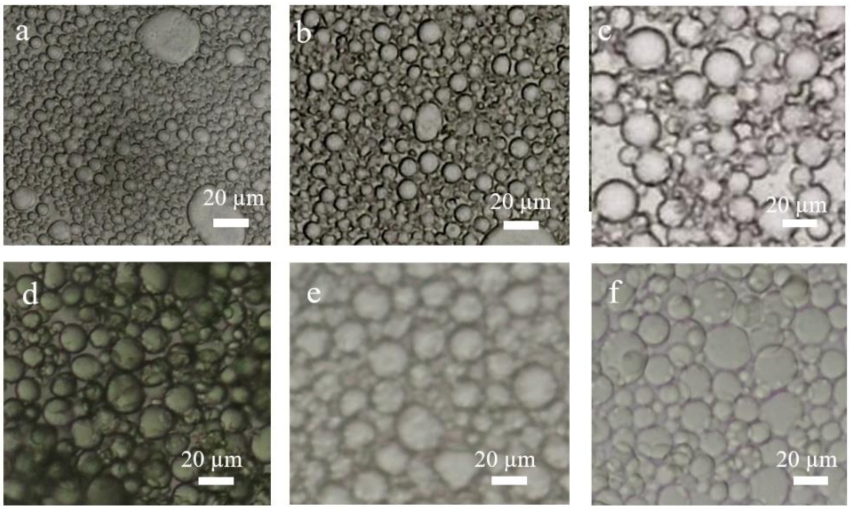

Figure. S3 Optical images of the silica wet gel microspheres of wg-SAMS-4.5-wet gel (a), wg-SAMS-3.5-wet gel (b), wg-SAMS-2.5-wet gel (c), teos-SAMS-4.5-wet gel (d), teos-SAMS-3.5-wet gel (e) , and teos-SAMS-2.5-wet gel (f).

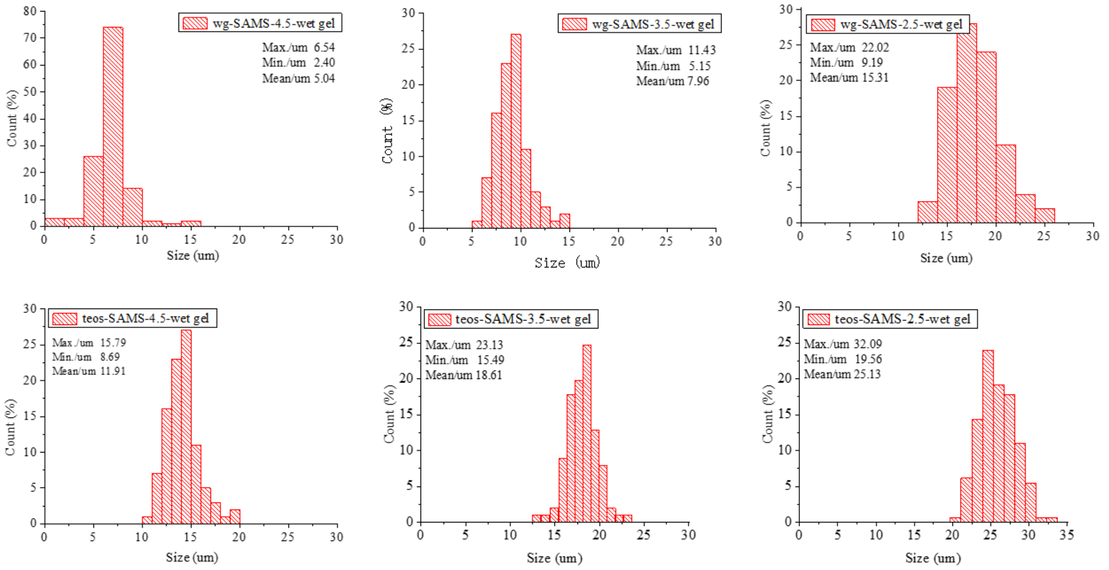

Figure. S4 Size distribution of silica wet gel microspheres.

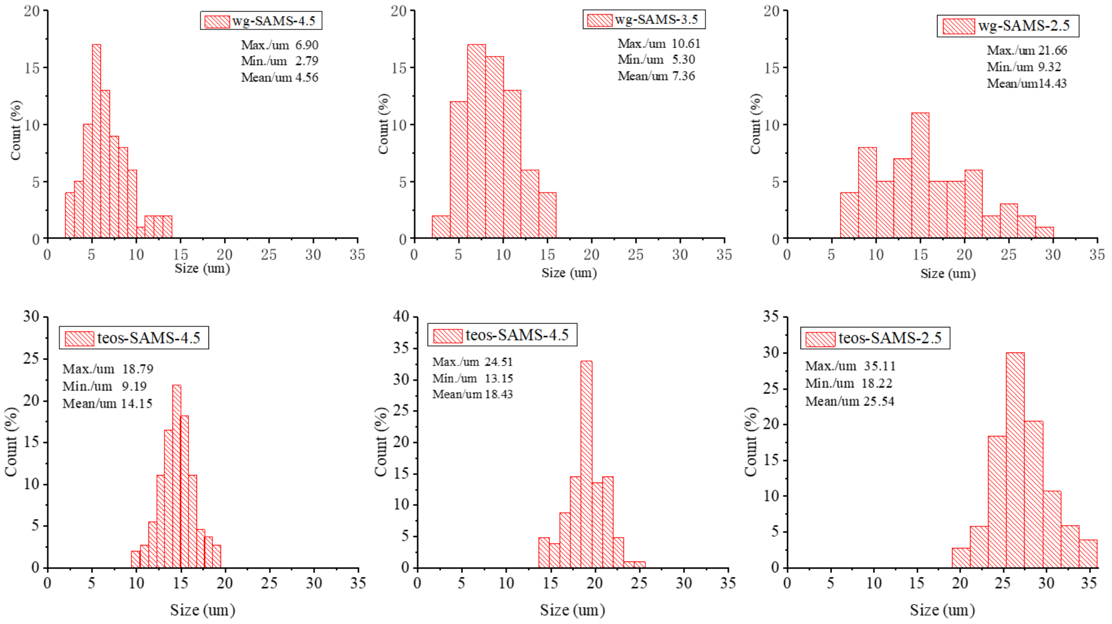

Figure. S5 Size distribution of silica aerogel microspheres.

S2. Adsorption–desorption isotherms

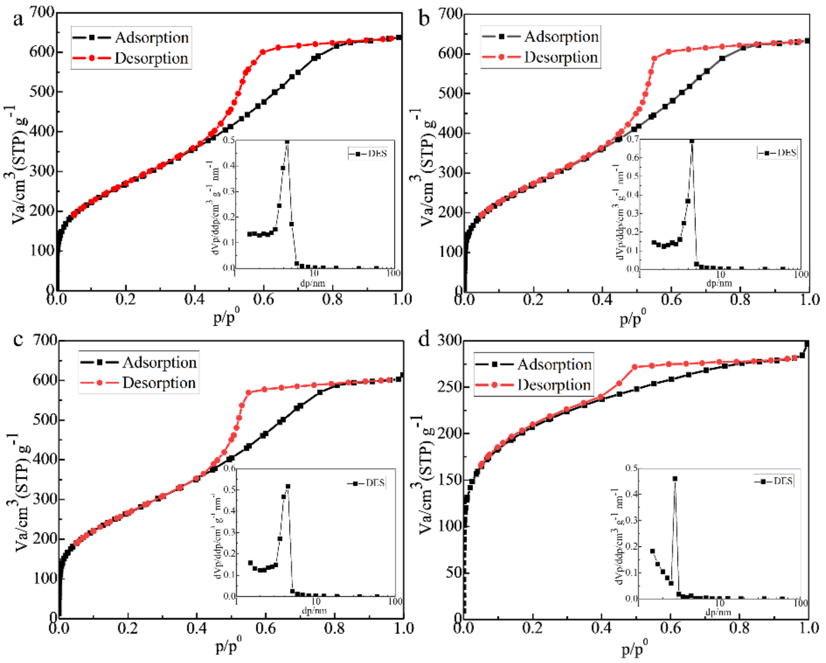

Figure. S6 Adsorption–desorption isotherms of silica aerogel microspheres of wg-SAMS-4.5 (a), wg-SAMS-3.5 (b), wg-SAMS-2.5 (c) and teos-SAMS-4.5 (d). Insets show the pore size distribution of the porous structure of the silica aerogel microspheres based on BJH (adsorption).

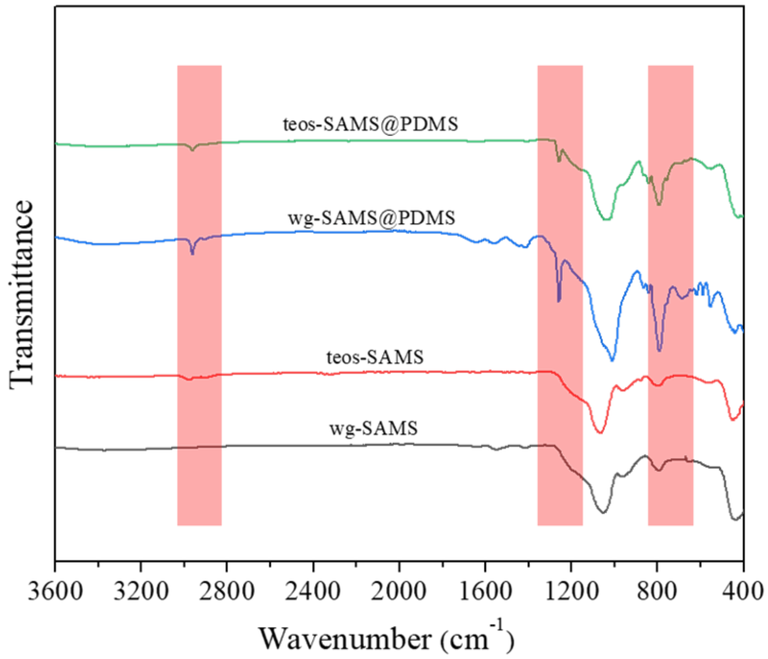

Figure. S7 FTIR spectra curves of aerogel specimens of teos-SAMS, wg-SAMS, teos-SAMS@PDMS and wg-SAMS@PDMS.

Table. S1 Physical properties of silica aerogel microspheres of wg-SAMS-4.5, wg-SAMS-3.5, wg-SAMS-2.5 and teos-SAMS-4.5.

samples	Specific Surface Area ($m^2 \cdot g^{-1}$)	Mean Pore Diameter (nm)
wg-SAMS-4.5	988.14	3.9918
wg-SAMS-3.5	997.15	3.9267
wg-SAMS-2.5	975.96	3.8511
teos-SAMS-4.5	745.49	2.4403
